# Localising the auditory N1m with event-related beamformers: localisation accuracy following bilateral and unilateral stimulation

**DOI:** 10.1038/srep31052

**Published:** 2016-08-22

**Authors:** Lauren Gascoyne, Paul L. Furlong, Arjan Hillebrand, Siân F. Worthen, Caroline Witton

**Affiliations:** 1 Aston Brain Centre, School of Life and Health Sciences, Aston University, Birmingham, B4 7ET, UK; 2 Sir Peter Mansfield Imaging Centre, Department of Physics, University of Nottingham, Nottingham , NG7 2QL , UK; 3 Department of Clinical Neurophysiology and Magnetoencephalography Center, VU University Medical Center, 1081 HV Amsterdam, the Netherlands

## Abstract

The auditory evoked N1m-P2m response complex presents a challenging case for MEG source-modelling, because symmetrical, phase-locked activity occurs in the hemispheres both contralateral and ipsilateral to stimulation. Beamformer methods, in particular, can be susceptible to localisation bias and spurious sources under these conditions. This study explored the accuracy and efficiency of event-related beamformer source models for auditory MEG data under typical experimental conditions: monaural and diotic stimulation; and whole-head beamformer analysis compared to a half-head analysis using only sensors from the hemisphere contralateral to stimulation. Event-related beamformer localisations were also compared with more traditional single-dipole models. At the group level, the event-related beamformer performed equally well as the single-dipole models in terms of accuracy for both the N1m and the P2m, and in terms of efficiency (number of successful source models) for the N1m. The results yielded by the half-head analysis did not differ significantly from those produced by the traditional whole-head analysis. Any localisation bias caused by the presence of correlated sources is minimal in the context of the inter-individual variability in source localisations. In conclusion, event-related beamformers provide a useful alternative to equivalent-current dipole models in localisation of auditory evoked responses.

The auditory N1-P2 (and its magnetic counterpart, the N1m-P2m) is a biphasic cortical response evoked by the onset of sound energy^1^,^2^, originating from auditory cortex^3^,^4^,^5^. The N1 component, in particular, is widely used in electrophysiological and neuromagnetic studies exploring the cortical organisation of responses to sound^6^, so its accurate localisation in magnetoencephalographic (MEG) data is important.

MEG source-localisation of the auditory N1m has traditionally been performed using equivalent current dipole-modelling approaches, which show good concordance with intracranial measures^4^. Beamformers are an alternative class of MEG source reconstruction approaches that can localise patterns of spectral power in brain activity during a selected time-frequency window, and allow for the localisation of multiple sources^7^. They are typically used for, and are most ideally-suited to, the localisation of induced, or non-phase-locked, changes in on-going cortical oscillatory rhythms^8^. However, in the form of event-related (ER) beamformers, they have successfully been applied to the localisation of evoked responses^9^,^10^. ER beamformers utilise weights derived from un-averaged MEG data to subsequently reconstruct sources associated with the evoked-change in spectral power in the averaged data. All beamformer methods have the benefit of requiring no *a-priori* assumptions about the number of sources^11^, and are highly effective in increasing the signal-to-noise ratio of the brain signals^12^,^13^.

However, there is evidence to suggest that beamformer methods are not appropriate for the localisation of auditory evoked responses, because they depend on the assumption of no linearly correlated sources within the brain for the duration of the analysis time-window^11^. In the auditory N1m-P2m complex, stimulus-locked activity occurs predominantly in the hemisphere contralateral to the ear of stimulation but is also present to a lesser extent in the ipsilateral cortex^14^,^15^,^16^, and this time-locked co-occurrence of bilateral evoked activity effectively results in a pair of correlated sources. The degree of correlation is increased when using diotic stimulation, which elicits large evoked responses in both hemispheres, yet such stimulation of each ear is unavoidable in studies exploring binaural processing^17^. The correlation between source time-series has been shown to cause some localisation bias e.g.^18^, and may introduce spurious sources^19^,^20^. In the present study, this inaccuracy is contrasted with inter-individual variability in source localisations when comparing data from monaural and diotic stimulation, conditions which differ in the extent of correlated activity across hemispheres.

Special beamformer implementations have been developed that reduce the impact of correlated sources on the accuracy of source reconstruction^18^,^20^,^21^,^22^,^23^. Another approach to combatting the effects of bilateral correlated sources is to use standard (e.g., manufacturer-supplied) beamformer software packages in a region-of-interest approach whereby data from the channels over the hemisphere contralateral to each ear of stimulation are analysed separately, thus excluding as much as possible the correlated ipsilateral activity^17^,^24^. For highly-correlated auditory steady-state responses, analysing channels from one hemisphere does not completely eliminate the bias introduced by a correlated source in the opposite hemisphere^18^, but the degree of correlation in a larger, more transient response such as the N1m, is considerably less. Therefore, this study aims to determine whether the single-hemisphere analysis approach is feasible for studies measuring the N1m-P2m complex. Specifically, we compare event-related beamformer analysis of the auditory N1m and P2m responses with traditional dipole-modelling, and explore the effects of different modes of beamformer analysis (whole-head and half-head) of data recorded during either monaural or diotic auditory stimulation, a contrast designed to manipulate the degree of correlation between sources in opposite hemispheres.

## Results

**Figure 1:**
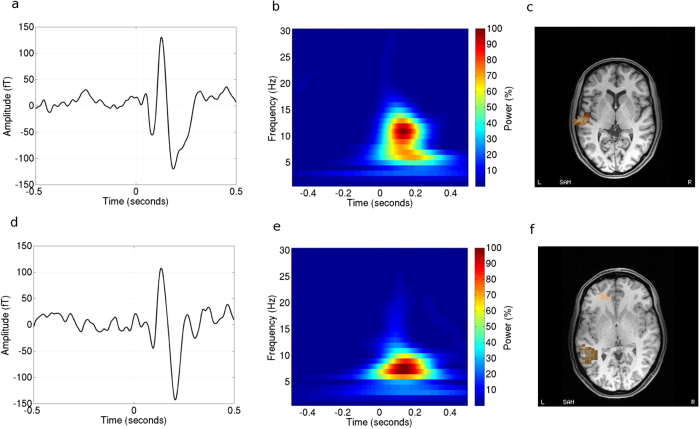
Representative data from two participants. (**a**,**d**) show averaged timeseries from a left hemisphere sensor taken at the peak of the posterior pole of the field pattern. (**b**,**e**) show wavelet spectrograms (using Morlet wavelets with a family parameter of 7 cycles) of the same timeseries. (**c**,**f**) show volumetric event-related beamformer images for the N1m response shown in each respective timeseries, for the alpha frequency band.

Figure 1 shows representative sensor data, taken from the peak of the left-hemisphere field pattern for two participants. The timeseries (Fig. 1a,d) show clear N1m and P2m peaks, and coincide with a single burst of spectral power (Fig. 1b,e) that shows some inter-individual variability in terms of frequency content. Fig. 1c,f show the event-related beamformer localisations of the N1m responses at source-level. Table 1 shows the mean and standard deviation latencies of the N1m and P2m evoked responses in the left and right hemispheres, for the 4–30 Hz frequency band in the diotic stimulation condition. For the N1m, latencies did not differ significantly in either hemisphere from those in the 1–30 Hz band used for dipole-modelling (*t*(10) = −0.54 and *t*(11) = −0.36. *p* > 0.05). For the P2m there was a small but significant difference in the right hemisphere (t(11) = 2.28, p = 0.043) but not the left (t(11) = 0.054, p > 0.05).

### Comparing ER beamformer and dipole localisation: N1m response

For the left hemisphere, the beamformer analysis yielded sources for 8 participants in the monaural condition, and 7 each in the diotic whole and half-head analyses. For the right hemisphere, 7 sources were obtained for monaural stimulation, 9 for half-head analysis of diotic data and 7 for whole-head analysis. Small differences in the numbers of acceptable sources across hemispheres and analysis methods were not significant (χ^2^, *p* > 0.05) given the sample-size used here.

**Figure 2:**
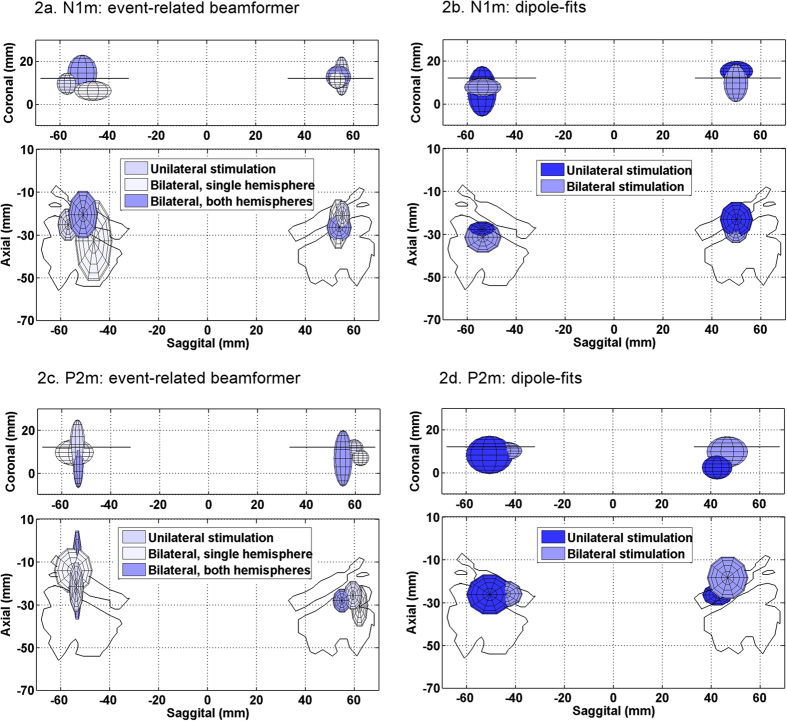
Source reconstructions for the N1m and P2m using the ER beamformer and dipole-fit approaches. (**a)** The mean and 95% confidence interval of the beamformer-reconstructed sources for each combination, plotted over the outline of auditory cortex (Heschl’s gyrus and planum temporale) in the 12 mm slice of the Talairach atlas for N1m. (**b)** The mean and 95% confidence interval for dipole-fit reconstructions for both stimulation conditions for N1m. (**c)**The mean and 95% confidence interval of the beamformer-reconstructed sources for each combination, plotted over the outline of auditory cortex (Heschl’s gyrus and planum temporale) in the 12 mm slice of the Talairach atlas for P2m. (**d)** The mean and 95% confidence interval for dipole-fit reconstructions for both stimulation conditions for P2m.

Figure 2a shows mean and 95% confidence volumes of the Talairach co-ordinates for the peak voxels in the left and right hemispheres, for the participants with activations within the acceptable boundaries. Importantly, all analyses yielded average source co-ordinates localised in auditory cortex. The mean co-ordinates of the ER beamformer source models were localised within, or overlapped with, the most anterior part of the temporal plane. Although the error volumes indicate inter-subject variability, this observation is broadly consistent with previous descriptions of the source of the N1m response and its electrophysiological counterpart^3^,^4^,^5^.

Confidence volumes for the dipole-fits are shown in Fig. 2b. For monaural stimulation, 9 acceptable dipole localisations were obtained in each hemisphere out of a possible 11; and for dichotic stimulation 7 were obtained in the left and 5 in the right hemisphere (no significant differences; χ^2^, *p* > 0.05). Consistent with previous observations (e.g.^25^,^26^,^27^) our N1m dipole-fits were more anterior in the right hemisphere, where confidence volumes crossed both Heschl’s gyrus and planum temporale, compared to the left hemisphere, where they fell entirely within planum temporale. This phenomenon was not observed for the ER beamformer localisations. However the 95% confidence volumes for dipole-fits overlap with those for the ER-SAM analyses. These confidence volumes are fairly large, indicating that there is considerable variation across subjects.

In considering the size of the confidence volumes for the dipole and ER beamformer source localisations, we can compare them to the inherent reliability of each method by conducting a split-half analysis. New datasets were computed including only the odd-numbered or even-numbered trials from the diotic stimulation condition and analysed using the ER beamformer and the dipole-fitting method. Comparing localisations for each pair of new datasets, pooling left and right hemispheres, gives an indication of the inherent reliability of each method. The mean distance in mm for x, y and z co-ordinates for SAM are 8.33, 8.98 and 5.14 with error volumes of 11.52, 5.11 and 3.89 respectively. The mean distance in mm for x, y and z co-ordinates for dipole fits are 11.27, 5.64 and 4.66 with error volumes of 7.21, 4.59 and 3.27 respectively. The two methods are very similar in deviation with the dipoles being very slightly more consistent. Intra-class correlations for the beamformer in the x, y, and z dimensions were 0.54, 0.99 and 0.67 with p-values ranging from 0.04 to <0.01. There are differences in the x and z directions but the y-direction appears to be stable. For the dipole analysis, ICC values were 0.85, 0.98 and 0.82, all p < 0.01, which is an almost perfect agreement. Thus the dipole analysis appears to have more inherent stability for this kind of data, particularly in the y-direction, although it should be noted that this analysis used half the number of trials in each analysis compared to our original analysis. These results indicate that the most uncertainty lies within the x-direction. The N1m response as a whole flows along the length of the planum temporale so it activates a relatively large area of cortex in this direction^28^. By picking the peak of activity we tried to choose the same phase of the response for each person, however individual differences in the source models could mean that the responses occur at slightly different phases, leading to differences in the peak locations within the x-direction.

### Comparing ER beamformer and dipole localisation: P2m response

In the left hemisphere, 9 participants showed acceptable sources in the monaural condition, 9 for diotic stimulation in the half-head analysis and 5 for the whole-head analysis. For the right hemisphere, the number of activations was 6, 7, and 5 respectively. Although the whole-head analysis of data from diotic stimulation yielded fewest sources, there was no statistically significant difference for the sample-size used here (χ^2^, *p* > 0.05).

Figure 2c shows mean locations and 95% confidence-intervals for each stimulus and ER-beamformer analysis condition for the P2m response. Mean sources all fell within auditory cortex, as for the N1m and as expected. Variations in localisation across stimulation and analysis conditions, which were most marked in the anterior-posterior axis, fell within the bounds of these error volumes. In the lateral-medial axis, mean locations fell within the lateral portion of HG and planum temporale. Dipole-fits for both stimulation conditions are shown in Fig. 2d, and, as for the N1m localisations, were consistent with the ER-beamformer data. Acceptable dipole-fits were achieved for 10 out of a possible 11 in the right hemisphere and 6 in the left hemisphere for the monaural stimulation condition; and 7 in the right hemisphere and 6 in the left hemisphere for bilateral stimulation. In the left hemisphere, the mean location of the P2 dipole fits fell in HG, unlike the dipole-fits for the N1m responses which localised on average to planum temporale. This trend for the P2m responses to lie anterior to N1m responses was observed in both hemispheres and is consistent with the literature^3^.

### Comparing half-head ROI analysis with whole-head analysis for diotic stimulation

No stimulation or analysis condition yielded activations in all 11 participants. When the data for diotic stimulation were analysed using all channels, there was no significant reduction in the number of sources yielded, compared to the analysis with only half the channels (χ^2^, *p* > 0.05). Tables 2 and 3 show the mean Talairach co-ordinates for the ER-beamformer conditions and the dipole fit localisations for the N1m and P2m respectively. Figure 2a,c do not show any trend for the half-head sensor data to yield more lateral localisations than the whole head sensor data as has previously been described for dipole models^29^. Paired analysis of response co-ordinates confirms that there is no significant difference (Wilcoxon signed ranks: left hemisphere, W = 18, p > 0.05, n = 6; right hemisphere, W = 8, p > 0.05, n = 5). Although the 95% confidence volumes for data from diotic stimulation analysed using all channels (Fig. 2b,d) appears to yield larger 95% confidence volumes than the same data analysed using only the channels over the hemisphere contralateral to stimulation (Fig. 2d) or data for monaural stimulation, analysed using all the channels (Fig. 2d), these differences were not statistically significant (Wilcoxon signed rank, p > 0.05).

## Discussion

By comparing results from both monaural and diotic stimulation, this study shows that the whole and half-head ER-beamformer can reliably localise both components of the auditory evoked response to auditory cortex, with similar accuracy at the group level as traditional dipole-modelling which can be considered the ‘gold standard’ (Fig. 2). The comparison illustrates that any bias that might be expected from the presence of correlated sources in opposite hemispheres does not have a marked effect on the beamformer results. The success-rate of the ER-beamformer was comparable to that of the more traditional dipole-fitting method, indicating that patterns of spectral power change that were localised by the beamformer were closely associated with the evoked response timeseries. This is consistent with the view that evoked responses emerge from a systematic reorganisation of ongoing cortical rhythms, both of which are measurable at the macroscopic level^30^. Overall, our results show that event-related beamformers do provide an appropriate method for localising transient auditory evoked responses, despite the theoretical problems associated with the presence of correlated sources in opposite hemispheres.

It is known that the N1m and P2m are not static responses, but rather reflect the dynamic activation of a series of sources within auditory cortex, including Heschl’s gyrus, Brodman area 22 which lies anterior to HG, and planum temporale^2^,^4^,^5^. Detailed MEG localisations of the N1m and P2m responses^3^ comply with the evidence from intracranial recordings^4^, showing that a large proportion of the activity in the post-100 ms component of the N1m originates from planum temporale, and that the P2m originates predominantly from a region anterior to this, in Heschl’s gyrus. Our mean dipole localisations showed a trend that is consistent with these known origins, especially for the left hemisphere, but our beamformer localisations did not. It is possible that the burst of spectral power (seen in Fig. 1), which forms the basis for the construction of the beamformer weights, is primarily associated with the N1m, which would have the effect of biasing the P2m localisations towards the planum temporale (i.e. towards the N1m sources). The time/frequency trade-off is a limitation of ER-beamformers in general, making the length of the time windows and the frequency bands chosen very important. The time windows used in this study were shorter than in some other work^31^, which should increase the detectability of a burst of spectral power falling within it, but depends on the assumption that each phase of the evoked response is associated with spectral power in the frequency band of interest: a potential limitation of this approach.

Similarly, the ER beamformer analysis failed to show the expected trend for right-hemisphere N1m sources to lie slightly more anterior than those in the left hemisphere^25^,^26^,^27^,^32^, although this right-hemisphere anterior shift was seen in our dipole localisations. Again, it is possible that these subtle dissociations between localisations using the two methods reflect a difference in the topographical distributions of the underpinning physiological signals being localised by each method, i.e. bursts of evoked spectral power versus peaks in timeseries amplitude. However it is not possible to rule out the effects of inter-subject variability with the relatively small sample-size used here. It is possible, but unlikely, that the differences between results from the two source localisation methods arises from differences in head model. The multi-sphere head model has been widely used with beamformers (e.g.^19^,^33^), and has been shown to provide more accurate source reconstructions than a single sphere model^34^ and comparable accuracy compared to BEM models^35^. Importantly, dipole fitting has inherently lower spatial resolution than beamforming (see Appendix in ref. 36), such that the effects of head modelling inaccuracies are less severe for dipole fitting than for beamformer analysis^36^. Here, we chose to use the optimal models available to us for each localization method.

Variability, and inaccuracy, in response localisation can result from a number of experimental factors affecting both source-modelling methods, including contamination of the average from physiological artefacts such as eye-blinks or EMG; co-registration errors; or movement during recording (where this is not compensated by software). There are also differences in the resolution bias of the dipole fit versus the beamformer technique, though the size of the confidence intervals would suggest that a smaller beamformer grid step size would not have resulted in a better match given the inter-subject variability. Individual factors such as differences in temporal-plane anatomy, including the presence of additional transverse gyri^5^ may contribute to differences in localisation when expressed on a standard atlas, such as the Talairach atlas as used here. Therefore, in summary, there are a large number of potential sources of inaccuracy in source localisation – in addition to differences in the underlying signal being analysed - which may have contributed to differences from results obtained using dipole models.

The localisations of the N1m and P2m in each hemisphere are consistently well defined in the literature, however there have been different reports of the degree of activation in the right region compared to the left region in response to contra-laterally presented stimuli^37^,^38^,^39^. Mis-localisation of the auditory source in either hemisphere could typically lead to an underestimation of the activity level at that source^40^. Full consideration of this issue is beyond the scope of the current report, however it is a valid concern for future work.

The second objective of this study was to determine whether diotic stimulation, as opposed to monaural stimulation, introduced any further bias into the ER-beamformer analysis, and to explore the effects of using a half-head, compared to whole-head analysis. To test this, we compared data from monaural stimulation (clicks presented to the right or left ear in separate recordings) to data from diotic stimulation that was analysed either with all the channels included, or from just the channels over the hemisphere contralateral to stimulation. We predicted that, should the effects of bilaterally correlated sources be significantly impairing beamformer localisation accuracy, we would see a greater degree of medial bias in the whole-head analysis than for the half-head analysis. Furthermore, it was possible that all the beamformer localisations would show a medial bias compared to the dipole localisations. In fact, as shown in Fig. 2, and a paired nonparametric test, there were no significant differences in localisation of the N1m or the P2m from the different stimulation or analysis conditions, or from the dipole localisations. The large confidence intervals on our group averages indicate that inter-subject heterogeneity is large, and the results suggest that any bias caused by the effects of bilateral phase-locked oscillations is small in the context of the individual variability (either natural heterogeneity or other sources of variability) in source localisations. This view is also compatible with previous analyses which have shown that beamformers are relatively robust to brief periods of correlation^41^,^42^, especially when the response being localised is of high signal-to-noise ratio^19^. Dipole-fitting methods themselves are not completely immune to the effects of cortical activity in the hemisphere ipsilateral to stimulation, especially in children where the head volume is smaller^29^, and our data suggest that beamformers perform no worse.

In conclusion, we have shown that event-related beamformers provide a source-modelling technique for the N1m that is as spatially accurate as the more traditional dipole fit approach. Source models derived using either method show inter-individual heterogeneity, and within this context the event-related beamformer offers an appropriate method for localising auditory evoked responses. Despite being susceptible to the influence of correlated sources under certain circumstances, any inaccuracies caused by the presence of bilateral auditory evoked responses are small in the context of large inter-individual anatomical variability under normal experimental conditions, leading to relatively large error volumes which may occlude any bias.

## Materials and Methods

### Participants

11 adults (7 females; age-range 26–71 years), with no reported neurological or audiological problems, took part in the study. The study was approved by the Aston University ethics committee, and was conducted in accordance with the Declaration of Helsinki. All participants gave informed, written consent.

### Stimuli

The stimuli were a train of 200 acoustic clicks of 1-ms duration, with an average inter-stimulus interval of 1200 ms, randomly jittered by up to plus or minus 100 ms. These clicks were delivered through echoless plastic tubing and foam ear-tips at a comfortable hearing level (approximately 70 dB SPL). Data were collected for two stimulus presentation conditions; monaural and diotic clicks. The monaural condition was run once for left-ear and once for right-ear stimulus presentation, so in total each participant yielded 3 datasets. The order of presentation for the three conditions was counterbalanced across participants.

### MEG data collection

Data were recorded using a 275-channel whole-head CTF MEG system (CTF Systems Inc., Port Coquitlam, BC, Canada) with a third-order synthetic gradiometer configuration^43^. During the recordings, participants were in the seated position, with their eyes open in a dimly-lit room, watching a silent video to maintain alertness. MEG data were sampled at 600 Hz using an antialiasing filter with a cutoff of 200 Hz, and comb-filtered to remove 50 Hz power-line artefact. The data were subdivided into epochs starting 500 ms before each click to 500 ms following each click. Each epoch was baseline-corrected by the mean amplitude of the 500-ms pre-stimulus period. The epochs were screened visually for physiological artefacts such as those arising from eye-blinks and muscle activity. A stringent approach to artefact removal then resulted in an average of 32 epochs per dataset being removed, leaving an average of 168 trials per dataset (SD = 16). Averaged evoked responses were computed and band-pass filtered according to the requirements of the analysis method, as described below. Evoked response latencies, shown in Table 1 for both the N1m and the P2m, were consistent with the literature^2^. For source-modelling, MEG data were spatially coregistered with the individual’s structural MRI using a modification of the surface-matching method described by^44^.

### ER Beamformer

ER beamformer source-models were computed using a method similar to that described by^45^, implemented using the CTF SAM (v. 5.11) software and Matlab2012a (The Mathworks Inc, Natick, MA, USA). We used the synthetic aperture magnetometry beamformer (SAM; ref. 46) which finds the optimal dipole orientation showing the maximum SNR at each voxel, providing an advantage over multidimensional beamformers^8^,^47^. No regularization was used. A full description of the method used to derive the ER beamformer timeseries can be found in ref. 45; in short, the virtual electrodes derived from the normalized beamformer weights are averaged across trials at each voxel and the power at a stimulus latency of interest can be visualised. The choice of time-frequency bins for analysis was based on pilot analysis of spectrograms of evoked activity in MEG sensors showing the peak evoked responses. Examples are shown in Fig. 1; there was considerable inter-subject variability in the frequency-content of spectrograms, with responses being generally broadband and dominated by lower frequencies. Based on this, we report data from analysis in one broad frequency band: 4–30 Hz. Note that due to the length of the time windows chosen, a wider bandwidth (i. e. 1–30 Hz) would not be possible without a detrimental effect on the data covariance estimation^45^. There was less inter- subject variability in the timing of responses than in their spectral content, so two time-windows were used for the computation of the data covariance matrix; each 200 ms in duration, and approximately centred on the N1m (0–200 ms) and P2m (50–250 ms) responses. This yielded 3 analyses per response, per hemisphere. We used a multi-sphere head model, derived from each individual’s outer skull surface as obtained from the MRI^35^.

To compute the ER beamformer images, the noise-normalised weights for each time-frequency window, derived from the un-averaged data, were applied to an averaged dataset which had been filtered in the relevant frequency-band. Images were computed for the data-point at the latency of the peak of the evoked response using a grid step size of 5 mm. Latencies used in this analysis had been recorded separately for each frequency-band to ensure that the peak amplitude was captured. The anatomical MRI was scaled to the Talairach co-ordinate system using MRI3dX software (v7.63) by manual identification of key landmarks^48^, enabling comparison of peak voxel co-ordinates between individuals. Activations were accepted if they fell broadly within, or close to, the superior surface of the temporal lobe, near or posterior to Heschl’s gyrus. They had to fall between co-ordinates of 17 to +24 mm in the Z direction (inferior-superior), between 0 and −60 mm in the X-direction (anterior-posterior), and were not restricted in the Y-direction (left-right) except that they had to fall within the expected hemisphere. The lack of restriction in the Y-direction is important given our planned analysis of expected bias in this dimension. Only the 5 largest volumetric peaks in the 3D ER beamformer image were considered as possible sources, because we expected auditory cortical activation to be the only significant task-related activity observed in this paradigm and peaks of smaller amplitude were likely to be spurious. Accepting peaks within the top 5, rather than the expected top 2, allowed us to retain valid activations that occurred in the presence of localisable artefacts such as eye movements (localising to the eyeballs) and electromyogram (localising to the neck or jaw area). It also allowed us to exclude activations falling outside of our ROI, which may occur as a result of correlated activity^23^.

### Equivalent current dipole-modelling

Dipole models were fitted for comparison with the ER beamformer source models. For each participant, a single-sphere volume conduction model was centred along the anterior-posterior dimension of the brain, with the radius adjusted to just encompass the surface of the temporal lobe in the coronal view. This approach ensured that the outline of the skull near the temporal lobes was well-described by the sphere, which would not be the case if the head-model had been fit to the whole skull. Data were band-pass filtered between 1 and 30 Hz, and dipoles were modelled using a least-squares minimisation method (CTF DipoleFit software v5.1.1), at the latency of the maximal response within the range of 70–140 ms (N1m) and 140–250 ms (P2m).

Modelling the source of the auditory evoked response using this method is challenging, because of the pair of mirror-image field patterns observed on the sensors, as well as a slight inter-hemispheric latency difference which is frequently observed^29^. For data from monaural stimulation, dipoles were modelled using only the MEG channels located over the hemisphere contralateral to stimulation. For the diotic condition, two dipoles were modelled simultaneously. When this method did not yield a source model for each hemisphere, a dipole was fit to the data from the sensors over the hemisphere yielding the best fit, and this was fixed in location and orientation before fitting the contralateral dipole, at which point the first dipole was refit. For the N1m response, the right dipole was fit first for 4 participants and the left for 2; for the P2m, the right dipole was fit first for 2 participants and the left for 5. Dipole co-ordinates were manually converted to Talairach co-ordinates based on the individual’s MRI, in the same way as for the ER beamformer co-ordinates.

### Statistical Analysis of Source Locations

For each stimulation and analysis condition, the Talairach co-ordinates for each participant’s peak acceptable activation or dipole location were averaged within the group, and 3-dimensional 95% confidence volumes were computed. Overlap between 95% confidence intervals for two conditions indicated the absence of a significant difference in localisation at the 5% alpha level although this does not include any correction for multiple comparisons.

The large number of conditions in the study (stimulus and analysis conditions, and frequency bands) inflates the likelihood of false-positive statistical findings. However stringent corrections for multiple comparisons are not appropriate, because many of the conditions are related; i.e., they are different analyses of the same data. Our approach therefore focused on looking for consistent patterns in the localisations of sources across conditions, based on the overlap of 95% confidence volumes and against two pre-specified research questions: (i) Do ER-beamformers yield the same localisations as equivalent current dipole-models, on average? (ii) Does the half-head analysis, using only sensors covering the hemisphere contralateral to stimulation, improve localisations for bilateral stimulation?

Mean and 95% confidence intervals for the activations were plotted over the outline of left and right auditory cortex (Heschl’s gyrus and planum temporale), traced from the 12 mm slice of the Talairach atlas^48^. Number of activations for different conditions were also compared where appropriate using a Chi-square test. All statistical analyses were performed in Matlab 2012a (The Mathworks Inc, Natick, MA, USA).

## Additional Information

**How to cite this article**: Gascoyne, L. *et al*. Localising the auditory N1m with event-related beamformers: localisation accuracy following bilateral and unilateral stimulation. *Sci. Rep.* **6**, 31052; doi: 10.1038/srep31052 (2016).

## Figures and Tables

**Table 1:** Latencies of the peak evoked responses (with standard deviation) during the 70–140 ms (N1) and 140–250 ms (P2) ranges, for the frequency bands used for the ER beamformer and dipole fit approaches.

Response	Frequency Band	Mean peak latency [msec]	
		Left Hemisphere	Right Hemisphere
N1	Theta, Alpha &Beta (4–30 Hz)	0.118 (10)	116 (16)
	Dipole-fits(1–30 Hz)	118 (12)	116 (17)
P2	Theta, Alpha, &Beta (4–30 Hz)	201 (19)	189 (40)
	Dipole-fits(1–30 Hz)	203 (21)	209 (14)

Latencies for the theta, alpha, and beta bands did not differ significantly (*p* > 0.05).

**Table 2:** Mean Talairach co-ordinates and 95% confidence intervals (in brackets) of the N1m response for ER-beamformer conditions in the 4–30 Hz band and the dipole fits in the 1–30 Hz band.

	Left hemisphere			Right hemisphere		
	X	Y	Z	X	Y	Z
Dipole–diotic stimulation	−53.71 (7.52)	−28.54 (5.79)	9.8 (8.86)	52.54 (4.53)	−27.39 (3.86)	17.54 (7.81)
Dipole–monaural stimulation(contralateral hemisphere)	−54.04 (5.88)	−27.79 (3.67)	5.86 (11.74)	49.74 (4.92)	−28.54 (5.79)	9.80 (8.86)
Half head ER SAM–diotic stimulation	−46.6 (7.54)	−32.77 (19.56)	6.23 (4.49)	53.37 (3.98)	−25.00 (12.08)	11.69 (4.45)
Whole head ER SAM–diotic stimulation	−50.95 (6.00)	−20.58 (11.48)	14.59 (8.40)	53.72 (5.05)	−26.54 (6.81)	12.83 (5.17)
Whole head ER SAM–monauralstimulation (contralateral hemisphere)	−57.08 (4.18)	−25.28 (7.84)	9.57 (4.99)	55.03 (3.03)	−20.91 (7.01)	13.17 (9.10)

**Table 3:** Mean Talairach co-ordinates of the P2m response for ER-beamformer conditions in the 4–30 Hz band and the dipole fits in the 1–30 Hz band.

	Left hemisphere			Right hemisphere		
	X	Y	Z	X	Y	Z
Dipole–diotic stimulation	−44.07 (6.82)	−26.02 (6.17)	10.12 (4.19)	42.49 (6.15)	26.44 (5.12)	2.41 (5.5)
Dipole–monaural stimulation(contralateral hemisphere)	−50.57 (9.54)	−26.25 (9.61)	8.28 (8.84)	46.64 (8.35)	−18.48 (10.27)	9.74 (7.13)
Half head ER SAM–diotic stimulation	−53.98 (7.53)	−24.19 (10.82)	12.06 (5.87)	53.56 (7.41)	−27.06 (12.66)	11.04 (4.42)
Whole head ER SAM–diotic stimulation	−56.78 (7.43)	−15.78 (9.89)	10.9 (7.19)	49.88 (7.58)	−21.14 (12.27)	11.64 (8.66)
Whole head ER SAM–monauralstimulation (contralateral hemisphere)	−54.4 (7.82)	−18.32 (14.5)	21.58 (7.39)	56.79 (4.03)	−21.3 (7.51)	8.24 (6.73)
